# Diabetic Retinopathy in Pregnancy: A Population-Based Study of Women with Pregestational Diabetes

**DOI:** 10.1155/2015/310239

**Published:** 2015-04-06

**Authors:** Aoife M. Egan, Lyle McVicker, Adrienne Heerey, Louise Carmody, Fiona Harney, Fidelma P. Dunne

**Affiliations:** ^1^Galway Diabetes Research Centre, National University of Ireland Galway and University Hospital Galway, Newcastle, Galway, Ireland; ^2^Department of Ophthalmology, National University of Ireland Galway and University Hospital Galway, Newcastle, Galway, Ireland

## Abstract

The aim of this observational study was to evaluate screening and progression of diabetic retinopathy during pregnancy in women with pregestational diabetes attending five antenatal centres along the Irish Atlantic seaboard. An adequate frequency of screening was defined as at least two retinal evaluations in separate trimesters. Progression was defined as at least one stage of deterioration of diabetic retinopathy and/or development of diabetic macular edema on at least one eye. Women with pregestational diabetes who delivered after 22 gestational weeks (*n* = 307) were included. In total, 185 (60.3%) had an adequate number of retinal examinations. Attendance at prepregnancy care was associated with receiving adequate screening (odds ratio 6.23; CI 3.39–11.46 (*P* < 0.001)). Among those who received adequate evaluations (*n* = 185), 48 (25.9%) had retinopathy progression. Increasing booking systolic blood pressure (OR 1.03, CI 1.01–1.06, *P* = 0.02) and greater drop in HbA1c between first and third trimesters of pregnancy (OR 2.05, CI 1.09–3.87, *P* = 0.03) significantly increased the odds of progression. A significant proportion of women continue to demonstrate retinopathy progression during pregnancy. This study highlights the role of prepregnancy care and the importance of close monitoring during pregnancy and identifies those patients at the highest risk for retinopathy progression.

## 1. Introduction

Deterioration of diabetic retinopathy during pregnancy is well described in women with pregestational diabetes mellitus [1–3]. This progression is influenced by multiple factors including the pregnancy itself, glycemic control before and during pregnancy, and the presence of preexisting retinopathy [4, 5]. Maternal medical complications including pregnancy-induced hypertension, diabetic nephropathy, and preeclampsia are also associated with progression of retinopathy [6, 7]. Unfortunately, study sample size has frequently limited the evaluation of additional risk factors and many studies predate the era of modern diabetes care in pregnancy which includes tight glycemic control and blood pressure management [1, 2, 8, 9]. Additionally, there is no data on screening rates within populations or on factors associated with receiving adequate retinal examinations during pregnancy.

The Atlantic Diabetes in Pregnancy (Atlantic DIP) initiative was established in 2005 and represents five antenatal centers along the Irish Atlantic seaboard, covering a population of 500,000 mixed urban and rural dwellers. The group offers women specialist-led, evidence-based care before, during, and after pregnancy and has significantly improved local outcomes in women with diabetes in pregnancy [10].

The aim of this study was to review the frequency of retinal examination during pregnancy in the Atlantic DIP cohort and examine maternal factors associated with receiving the optimal number of examinations. Additionally, we documented the progression of diabetic retinopathy during pregnancy and factors associated with this progression.

## 2. Methods

### 2.1. Study Design and Population

This study was designed as an observational study of retinopathy status during pregnancy in women with pregestational diabetes. Research ethics committee approval was obtained and women were recruited between September 2006 and December 2012. Women with singleton pregnancies who provided informed consent were included. As per previous studies, women were classified as having pregestational diabetes on the following basis: (1) an established diagnosis of type one or type two diabetes mellitus prior to conception or (2) glycosylated hemoglobin (HbA1c) greater than 6.5% in the first trimester [11]. Data were collected from study entry until 12 weeks postpartum using an optimized digital database, namely, DIAMOND (Hicom).

### 2.2. Procedures

Prior to pregnancy, during annual review appointments, all women were advised regarding the need to plan pregnancy and were offered the opportunity to attend a dedicated, prepregnancy service. During pregnancy, each woman received standard advice on diet and exercise along with a dietician review. Education was provided on self-directed glucose monitoring and each woman was advised on glycemic targets. Women were reviewed on a fortnightly basis and telephoned on a weekly basis. Insulin was introduced (in the setting of type two diabetes mellitus) or adjusted if home glucose readings were outside the following ranges on more than three consecutive days: fasting glucose 5.0 mmol/L or a 2-hour postprandial reading of 7.0 mmol/L. Oral hypoglycemic agents were not used during the study period. Retinal screening should occur at least twice during pregnancy in separate trimesters and if established retinopathy is present, then retinal examination should take place more frequently. For the purposes of this study, we accepted at least two retinal evaluations in separate trimesters as adequate. During the study period, retinal examination took place in the locality of each antenatal center and results were forwarded to the respective center. At each visit, visual acuity was measured bilaterally using the Snellen chart. The pupils were then dilated with tropicamide 1% and ophthalmological examinations were performed using a two-field photography system. These images were reviewed by an accredited retinal grader. If photo screening was not performed or if the images were abnormal, an experienced ophthalmologist performed an eye examination. Progression was defined as at least one stage of deterioration of diabetic retinopathy and/or development of diabetic macular edema on at least one eye. Grading standards as outlined by the National Screening Committee in the United Kingdom were followed (Table 1) [12]. Antihypertensive therapy was initiated when blood pressure was ≥135/85 mmHg. Labetalol was the first-choice antihypertensive agent, followed by methyldopa when needed.

### 2.3. Statistical Analysis

Data were analyzed using Statistical Package for the Social Sciences (SPSS) version 20.0 (IBM). Hypothesis testing was performed on the data of equal variance and normal distribution using an unpaired Student's *t*-test. The Mann-Whitney *U* test was used as the equivalent nonparametric test. Chi squared analysis was used to compare sample proportions. Binary logistic regression was utilized to assess the association of multiple covariates with receipt of adequate retinal evaluations and the progression of retinopathy. Data are expressed as means ± standard deviation (SD) or the mean, adjusted odds ratios (aORs), and 95% confidence intervals (CI). Statistical significance was accepted when the 95% CI did not contain one (regression analyses/ratios) or zero (multiple group comparisons/means). The significance level (*α*) was accepted when <0.05 for two-tailed analyses.

## 3. Results

We identified 307 women with pregestational diabetes who delivered after 22 gestational weeks from the Atlantic DIP database. This cohort comprised 208 (67.8%) with type one diabetes and 99 (32.2%) with type two diabetes. The majority of women were of Caucasian ethnicity (*n* = 278, 90.6%). There were 296 (96.4%) live births and 11 (3.6%) stillbirths.

Overall, 217 (70.7%) of 307 women had retinal evaluation at least once during pregnancy and 185 (60.3%) had an adequate number of examinations. Of the 32 women who had screening on one occasion only, 29 (90.6%) had no retinopathy and 3 (9.4%) had background retinopathy.  Table 2 outlines the characteristics of those who received an adequate number of examinations versus those who did not. Those who received an adequate number of retinal evaluations were older (32.9 ± 5.3 versus 31.5 ± 5.4 years, *P* = 0.02) and a higher proportion were of Caucasian ethnicity (94.1% versus 85.2%, *P* = 0.01). There was no difference in gravida, parity, first or third trimester HbA1c, or smoking status between the two groups. Among women with type one diabetes, 64.4% received an adequate number of examinations, compared with 51.5% of women with type two diabetes. A higher proportion of women who received appropriate screening had attended prepregnancy care (58.4% versus 17.2%, *P* < 0.001) and taken folic acid preconceptually (69.7% versus 54.1%, *P* = 0.001). A higher proportion of women received adequate examinations in the years 2009 to 2012 compared with the years 2006 to 2008 (74.1% versus 25.9%).

A logistic regression model was completed to further examine maternal factors associated with receiving an adequate frequency of ophthalmological evaluation in pregnancy. The results are outlined in Table 3 and they identified attendance at prepregnancy care as the only maternal factor significantly associated with receiving appropriate screening (odds ratio 6.23; CI 3.39–11.46 (*P* < 0.001)).

On evaluation of those patients who received two or more retinal evaluations (*n* = 185), 48 (25.9%) had retinopathy progression during pregnancy. This represents 15.6% of all included patients (*n* = 307).  Table 4 further outlines the characteristics of the women. A higher proportion of women with type one diabetes progressed compared with type two diabetes (31.3% versus 11.7%, *P* = 0.001). Those women who demonstrated progression had, on average, a longer duration of diabetes (14.43 ± 8.42 versus 9.79 ± 8.36 years, *P* < 0.001). Although there was a higher frequency of preeclampsia in the group that experienced progression, this was not significant (14.6% versus 12.4%, *P* = 0.80). However, those who did progress had a significantly higher systolic blood pressure at their initial antenatal visit (128.6 ± 18.0 versus 122.1 ± 13.0 mmHg, *P* = 0.03). Additionally, first trimester HbA1c was higher (7.67 ± 1.62 versus 7.03 ± 1.39%, *P* = 0.01) and the drop in HbA1c between first and third trimesters was greater (1.38 ± 1.33 versus 0.74 ± 0.90%, *P* = 0.004) among those women who had disease progression. There was no significant difference in rates of excessive gestational weight gain, smoking status, or body mass index between the two groups.

Logistic regression analysis revealed that increasing systolic blood pressure at booking (OR 1.03, CI 1.01–1.06, *P* = 0.02) and greater drop in HbA1c between first and third trimesters of pregnancy (OR 2.05, CI 1.09–3.87, *P* = 0.03) significantly increased the odds of retinopathy progression. Duration of diabetes, diabetes type, and first trimester HbA1c were not associated with increased odds of progression and these results are outlined in Table 5.

Baseline retinal findings are outlined in Table 4. Of those women who developed progression (*n* = 48), 32 (66.7%) had no retinopathy at baseline. A total of 26 (54.2%) women progressed from no retinopathy to level 1 (background) retinopathy only. A further 7 (14.6%) progressed from level 1 to level 2 (preproliferative) retinopathy and 6 women (12.5%) progressed to level 3 (proliferative) retinopathy and required laser therapy. Two women in the latter group had also received prepregnancy laser therapy. Finally, 6 (12.5%) women developed mild maculopathy and three (6.3%) experienced a worsening of preexisting maculopathy and required laser therapy. Among the group with maculopathy development (*n* = 9), 4 (44.4%) women received prepregnancy laser therapy.

## 4. Discussion

In an unselected population of women with pregestational diabetes, we demonstrate that 60.3% had an adequate number of ophthalmological examinations during pregnancy. Attendance at prepregnancy care was strongly associated with receiving adequate retinal evaluation in the subsequent pregnancy. Despite intensive glycemic control and antihypertensive therapy as required, progression of retinopathy was observed in 14% of the total group and in 26% of those who had more than one retinal examination during pregnancy.

Recommendations for retinopathy screening and management in pregnancy vary significantly. The American Diabetes Association advises an eye examination in the first trimester with close follow-up throughout pregnancy [13]. The National Institute for Health and Clinical Excellence (NICE) in the United Kingdom recommends retinal assessment following the first antenatal clinic appointment and again at 28 weeks if the first assessment is normal. If any diabetic retinopathy is present, an additional retinal assessment should be performed at 16–20 weeks [14]. For the purposes of this study, we accepted a minimum of two retinal evaluations in separate trimesters in accordance with local guidelines. Unfortunately, despite the existence of these guidelines, there was no retinal evaluation in 35% of cases and just one evaluation in a further 9%. While many studies in the area of diabetic retinopathy in pregnancy include selected patients with complete ophthalmological evaluations only [1, 15], a study of women with type two diabetes reported that only 73% had the available ophthalmological examinations [2]. In relation to this current study, there is not an automatic recall system for retinal evaluation in our antenatal centers and each woman must be referred individually. It is the opinion of the authors that an automatic, standardized system of follow-up as demonstrated by Hampshire et al. would improve screening and follow-up rates [4]. The majority of women who had just one eye examination were retinopathy-free and the lack of follow-up in later pregnancy may reflect a perceived “minimal risk” on behalf of the patient and health care provider with more focus being placed on those patients with established retinopathy.

Another interesting observation presented herein is the association between attendance at a prepregnancy care program and adequate retinal assessment in the subsequent pregnancy. It is reasonable to assume that the educational component of the program informs women of recommended intervals for ophthalmology review during pregnancy and these women are more likely to ensure they receive and attend appointments. The increased attendance at prepregnancy care undoubtedly explains the higher proportion of women taking prepregnancy folic acid in the group who received adequate eye assessments during pregnancy. Unfortunately, we do not have information regarding the exact timing of prepregnancy care and levels of metabolic control and blood pressure at the time of attendance. Although there was no significant difference in progression of retinopathy between patients attending prepregnancy care and those who did not, improved metabolic control just before pregnancy may have influenced retinopathy progression during the subsequent pregnancy. Finally, the higher rates of adequate screening in the latter four years of the study reflect improvements in clinical care delivery as the Atlantic DIP program became established.

This study highlights the ongoing risk of retinopathy progression during pregnancy particularly among women with type one diabetes. Rasmussen et al. evaluated 80 patients with type two diabetes and observed progression in 14% [2]. Vestgaard et al. evaluated 102 women with type one diabetes and noted progression in 27% [6]. These studies did not find an association between glucose control and progression of retinopathy but this may be due to very tight prepregnancy glycemic control or a type-two error due to a lesser number of included subjects. However, our observations reinforce other published works that noted both significant and nonsignificant trends towards progression of retinopathy in the setting of a greater drop in HbA1c during the pregnancy [5, 15, 16]. While the third trimester HbA1c was similar between groups that did and did not progress in our study, the first trimester value was on average 0.5% higher in the group that developed retinopathy progression. The importance of prepregnancy glycemic optimization should be highlighted as it is associated with a tendency toward less progression of retinopathy compared with waiting until pregnancy is confirmed in type one diabetes [2, 15]. In the setting of an unplanned pregnancy with poor glycemic control, the authors believe that glycemic control should be prioritized and appropriately optimized as the long-term consequences of poor glycemic control during the pregnancy appear to outweigh those of retinopathy progression [10, 15, 17, 18]. This issue has also received attention in studies involving a more general diabetes population. For example, although early worsening of diabetic retinopathy was noted in a higher proportion of those assigned to intensive treatment in the Diabetes Control and Complications Trial (DCCT), the long-term benefits of intensive insulin treatment greatly outweighed the risks of this early worsening [19]. The association between retinopathy progression and systolic blood pressure at booking is not unexpected as hypertensive disorders of pregnancy and indeed higher systolic blood pressure are factors known to negatively influence retinopathy [7, 20, 21].

In relation to the complication severity, two-thirds of women who experienced retinopathy progression developed background retinopathy only and no women with a normal retinal examination during trimester one developed sight-threatening disease or required laser therapy. All women who developed sight-threatening disease had significant changes identified at baseline. These findings are reassuring, particularly as Hellstedt et al. demonstrated a regression of mild retinopathy postpartum in a cohort of women with type one diabetes [22]. A limitation of the study is that we do not have postpartum evaluations to determine the longer-term progression of retinopathy. However, Arun and Taylor studied women with type one diabetes for 5 years after delivery and concluded that pregnancy is not associated with postpartum worsening of retinopathy [17]. Additionally, in the Pittsburgh EDC pregnancy study, it was observed that the overall prevalence of retinopathy in women with prior pregnancy was similar to that of matched nulliparous women [18].

Overall, this was a robust, nested cohort analysis performed retrospectively with data managed prospectively within the Atlantic DIP database. Although the observational study design has inherent limitations including the potential influence of unmeasured covariates such as additional medications, we have used robust statistical methods to evaluate rates of retinopathy progression and employed regression analysis to demonstrate factors associated with disease progression and adequate retinal evaluation during pregnancy. The management of the patients included in this study is a reflection of real-life clinical practice and involves patients with both type one and type two diabetes with varying durations of disease. The findings are externally valid particularly in relation to other predominantly Caucasian populations.

## 5. Conclusions

In summary, with the establishment of a structured antenatal care program, more women are receiving an adequate number of retinal examinations during pregnancy. The introduction of an automatic recall system has the potential to improve service delivery. A significant proportion of women continue to experience deterioration in retinopathy during pregnancy and this validates the need for close follow-up. Finally, the importance of prepregnancy care to fully inform women of the need for more frequent retinal assessments during pregnancy and allow preconceptual optimization of glycemic control and blood pressure should be emphasized. These results will assist the health care professional design and provid high quality antenatal care for women with pregestational diabetes mellitus.

## Figures and Tables

**Table 1:** Classification of diabetic retinopathy [12].

Grade	Description	Lesion
R0	No retinopathy	No apparent retinopathy

R1	Background retinopathy	Microaneurysm(s) Retinal haemorrhage(s) ± any exudate

R2	Preproliferative retinopathy	Venous beading, venous loop, or reduplication Intraretinal microvascular abnormality (IRMA) Multiple deep, round, or blot haemorrhages

R3	Proliferative retinopathy	New vessels on disc (NVD) New vessels elsewhere (NVE) Preretinal or vitreous haemorrhage Preretinal fibrosis ± tractional retinal detachment

M0	No maculopathy	No apparent maculopathy

M1	Maculopathy present	Exudate within 1 disc diameter (DD) of the centre of the fovea Circinate or group of exudates within the macula Retinal thickening within 1 DD of the centre of the fovea (if stereo available) Any microaneurysms or haemorrhage within 1 DD of the centre of the fovea only if associated with a best VA of ≤6/12 (if no stereo)

**Table 2:** Characteristics of women who received an adequate number of retinal examinations versus those who did not (*n* = 307).

	Adequate number of retinal examinations	Inadequate number of retinal examinations	*P* value
*N* (%)	185 (60.3%)	122 (39.7%)	
Type 1 diabetes	134 (72.4%)	74 (60.7%)	
Type 2 diabetes	51 (27.6%)	48 (39.3%)	
Age (years)	32.9 ± 5.3	31.5 ± 5.4	0.02
Gravida	2.4 ± 1.5	2.8 ± 2.2	0.16
Parity	0.9 ± 1.1	1.0 ± 1.3	0.13
Caucasian	174 (94.1%)	104 (85.2%)	0.01
Diabetes duration (years)	11.28 ± 5.68	9.25 ± 5.77	0.007
Nonsmokers	158 (85.4%)	110 (90.2%)	0.29
Attendance at prepregnancy care	108 (58.4%)	21 (17.2%)	<0.001
Folic acid	129 (69.7%)	66 (54.1%)	0.001
1st trimester HbA1c % (mmol/mol)	7.20 ± 1.48 (55.0 ± 4.5)	7.06 ± 1.63 (54.0 ± 4.6)	0.47
3rd trimester HbA1c % (mmol/mol)	6.28 ± 0.88 (45.0 ± 2.6)	6.37 ± 0.96 (46.0 ± 2.9)	0.43
Years 2006–2008	48 (25.9%)	84 (68.9%)	
Years 2009–2012	137 (74.1%)	38 (31.1%)	

Data expressed as mean ± standard deviation (SD), number of patients, and % of group.

**Table 3:** Maternal factors associated with receiving appropriate retinal evaluation during pregnancy.

	Odds ratio	Confidence interval	*P* value
Age	1.02	0.97–1.08	0.38
Ethnicity	0.71	0.27–1.85	0.48
Diabetes type	0.95	0.46–1.98	0.89
Diabetes duration	1.03	0.99–1.07	0.15
Attendance at prepregnancy care	6.23	3.39–11.46	<0.001
Folic acid use	0.97	0.56–1.67	0.97

**Table 4:** Women who received appropriate screening (*n* = 185). Characteristics of those who demonstrated retinopathy progression compared with those who did not.

	No progression	Progression	*P* value
*N* (%)	137 (74.1%)	48 (25.9%)	
Diabetes type 1	92 (67.2%)	42 (87.5%)	
Diabetes type 2	45 (32.8%)	6 (12.5%)	
Age (years)	32.64 ± 5.35	33.60 ± 5.15	0.28
Caucasian ethnicity	128 (93.4%)	46 (95.8%)	0.54
Parity	0.99 ± 1.16	0.73 ± 1.05	0.09
Gravida	2.44 ± 1.54	2.21 ± 1.53	0.17
Body mass index (kg/m^2^)	28.78 ± 6.32	27.50 ± 5.30	0.35
Prepregnancy care	82 (59.9%)	26 (54.2%)	0.49
Folic acid	97 (70.8%)	32 (66.7%)	0.59
Diabetes duration (years)	9.79 ± 8.36	14.43 ± 8.42	<0.001
Excessive weight gain in pregnancy	76 (55.5%)	29 (60.4%)	0.88
1st trimester	7.03 ± 1.39	7.67 ± 1.62	0.01
HbA1c (%)	(53.0 ± 4.2)	(60.0 ± 4.9)	
3rd trimester	6.27 ± 0.93	6.29 ± 0.70	0.89
HbA1c (%)	(45.0 ± 2.8)	(45.0 ± 2.1)	
Change in HbA1c between 1st and 3rd trimester (%)	0.74 ± 0.90 (9.6 ± 11.7)	1.38 ± 1.33 (17.9 ± 17.3)	0.004
Preeclampsia	17 (12.4%)	7 (14.6%)	0.80
Systolic blood pressure at booking (mmHg)	122.1 ± 13.0	128.6 ± 18.0	0.03
Diastolic blood pressure at booking (mmHg)	72.89 ± 10.3	76.0 ± 9.4	0.73
Nonsmoker	118 (86.1%)	40 (83.4%)	0.63
Baseline retinal findings			
R0 (no retinopathy)	82 (59.9%)	32 (66.7%)	
R1 (background)	33 (24.1%)	9 (18.8%)	
R2 (preproliferative)	6 (4.4%)	4 (8.3%)	
R3 (proliferative)	10 (7.3%)	0 (0%)	
Maculopathy	6 (4.4%)	3 (6.3%)	

Data expressed as mean ± standard deviation (SD), number of patients, and % of group.

**Table 5:** Factors associated with retinopathy progression.

	Odds ratio	CI	*P* value
Duration of diabetes	1.04	0.99–1.10	0.12
Diabetes type	0.47	0.15–1.54	0.21
1st trimester HbA1c	0.83	0.53–1.30	0.42
HbA1c reduction between 1st and 3rd trimester	2.05	1.09–3.87	0.03
Systolic blood pressure at booking	1.03	1.01–1.06	0.02

## References

[1] Klein B. E. K., Moss S. E., Klein R. (1990). Effect of pregnancy on progression of diabetic retinopathy. *Diabetes Care*.

[2] Rasmussen K. L., Laugesen C. S., Ringholm L., Vestgaard M., Damm P., Mathiesen E. R. (2010). Progression of diabetic retinopathy during pregnancy in women with type 2 diabetes. *Diabetologia*.

[3] Omori Y., Minei S., Testuo T., Nemoto K., Shimizu M., Sanaka M. (1994). Current status of pregnancy in diabetic women. A comparison of pregnancy in IDDM and NIDDM mothers. *Diabetes Research and Clinical Practice*.

[4] Hampshire R., Wharton H., Leigh R., Wright A., Dodson P. (2013). Screening for diabetic retinopathy in pregnancy using photographic review clinics. *Diabetic Medicine*.

[5] Chew E. Y., Mills J. L., Metzger B. E. (1995). Metabolic control and progression of retinopathy. The Diabetes in Early Pregnancy Study. National Institute of Child Health and Human Development Diabetes in Early Pregnancy Study. *Diabetes Care*.

[6] Vestgaard M., Ringholm L., Laugesen C. S., Rasmussen K. L., Damm P., Mathiesen E. R. (2010). Pregnancy-induced sight-threatening diabetic retinopathy in women with Type 1 diabetes. *Diabetic Medicine*.

[7] Lövestam-Adrian M., Agardh C., Aberg A., Agardh E. (1997). Pre-eclampsia is a potent risk factor for deterioration of retinopathy during pregnancy in type 1 diabetic patients. *Diabetic Medicine*.

[8] Dibble C. M., Kochenour N. K., Worley R. J., Tyler F. H., Swartz M. (1982). Effect of pregnancy on diabetic retinopathy. *Obstetrics & Gynecology*.

[9] Price J. H., Hadden D. R., Archer D. B., Harley J. M. G. (1984). Diabetic retinopathy in pregnancy. *British Journal of Obstetrics & Gynaecology*.

[10] Owens L. A., Avalos G., Kirwan B., Carmody L., Dunne F. (2012). ATLANTIC DIP: closing the loop: a change in clinical practice can improve outcomes for women with pregestational diabetes. *Diabetes Care*.

[11] Egan A. M., Dennedy M. C., Al-Ramli W., Heerey A., Avalos G., Dunne F. (2014). ATLANTIC-DIP: excessive gestational weight gain and pregnancy outcomes in women with gestational or pregestational diabetes mellitus. *Journal of Clinical Endocrinology and Metabolism*.

[12] UK National Screening Committee (2009). *Essential Elements in Developing a Diabetic Retinopathy Screening Programme*.

[13] American Diabetes Association (2013). Standards of medical care in diabetes—2013. *Diabetes Care*.

[14] http://www.nice.org.uk/guidance/cg63/chapter/guidance.

[15] The Diabetes Control and Complications Trial Research Group (2000). Effect of pregnancy on microvascular complications in the diabetes control and complications trial. *Diabetes Care*.

[16] Temple R. C., Aldridge V. A., Sampson M. J., Greenwood R. H., Heyburn P. J., Glenn A. (2001). Impact of pregnancy on the progression of diabetic retinopathy in Type 1 diabetes. *Diabetic Medicine*.

[17] Arun C. S., Taylor R. (2008). Influence of pregnancy on long-term progression of retinopathy in patients with type 1 diabetes. *Diabetologia*.

[18] Hemachandra A., Ellis D., Lloyd C. E., Orchard T. J. (1995). The influence of pregnancy on IDDM complications. *Diabetes Care*.

[19] The Diabetes Control and Complications Trial Research Group (1998). Early worsening of diabetic retinopathy in the diabetes control and complications trial. *Archives of Ophthalmology*.

[20] Rosenn B., Miodovnik M., Kranias G. (1992). Progression of diabetic retinopathy in pregnancy: association with hypertension in pregnancy. *American Journal of Obstetrics & Gynecology*.

[21] Axer-Siegel R., Hod M., Fink-Cohen S. (1996). Diabetic retinopathy during pregnancy. *Ophthalmology*.

[22] Hellstedt T., Kaaja R., Teramo K., Immonen I. (1997). The effect of pregnancy on mild diabetic retinopathy. *Graefe's Archive for Clinical and Experimental Ophthalmology*.

